# Patient Experiences of Patient‐Initiated Brief Admission in Psychiatric Care: A Systematic Review

**DOI:** 10.1111/inm.13457

**Published:** 2024-10-27

**Authors:** Emma Värnå, Jonas Nederman, Erika A. Saliba‐Gustafsson, Joachim Eckerström

**Affiliations:** ^1^ Department of Health Care Sciences Marie Cederschiöld University Stockholm Sweden; ^2^ Department of Clinical Neuroscience, Centre for Psychiatry Research Karolinska Institutet & Stockholm Health Care Services Region Stockholm Sweden; ^3^ Division of Nursing, Department of Neurobiology Care Sciences and Society, Karolinska Institutet Stockholm Sweden

**Keywords:** brief psychiatric hospitalisation, empowerment, mental health services, patient participation, qualitative research, thematic synthesis

## Abstract

Patient‐initiated brief admission (PIBA) is an innovative psychiatric care intervention that gives patients the autonomy to initiate a short admission (approximately 1–3 days) to psychiatric inpatient care. This intervention is structured around a mutual agreement between the patient and their care provider that outlines the specific structure and content of their care. Unlike regular psychiatric admissions, healthcare professionals do not review the patient's decision for admission during PIBA. Similar interventions have been developed globally to reduce the need for long inpatient admissions and compulsory care by enhancing patient autonomy, promoting active participation in care, and empowering patients to recognise early signs of mental health deterioration. The objective of this systematic review was to explore the experiences of PIBA among individuals with mental health disorders. A systematic review was conducted using qualitative articles sourced from the PubMed, CINAHL, and PsycINFO databases. A total of thirteen original articles were included in the review, encompassing 186 patients. Research demonstrates that PIBA significantly impacts patients' care experiences in various ways. Access to PIBA gives patients the opportunity to take a break from daily stressors, which has proven significant in interrupting the cycle of worsening symptoms and negative thoughts. Furthermore, when the care environment is characterised by trust and respect, patients experience an increased sense of freedom, which contributes to a more effective recovery process. PIBA provides patients with a sense of safety and offers the possibility of a more functional daily life. Healthcare professionals'attitude and care provision also significantly influences patients' experiences. Central to a positive patient experience are a warm reception, attentiveness, and active listening. PIBA can not only change patients' perceptions of healthcare but, more importantly, fosters a transformative view of themselves as active participants in their own well‐being. Knowledgeable healthcare professionals are crucial for the successful implementation of this intervention. By offering dignity and warmth alongside safety, PIBA addresses a critical gap in patient mental health care.

## 1 Introduction

In 2015, a literature review examining users' experiences of psychiatric care services worldwide uncovered a range of positive and negative interactions within healthcare settings, with negative experiences being more prevalent (Newman et al. 2015). Positive experiences were characterised by supportive and empathetic staff, access to therapy and medication, and notable improvements in well‐being. In contrast, negative experiences included poor communication and collaboration with staff, lack of continuity, and limited access to care. Given these varied experiences, a transformation in the paradigm of patient care is crucial (Newman et al. 2015). A more holistic vision that recognises patients as unique individuals with their own will, thoughts, and feelings, combined with care addressing their psychosocial, emotional, existential, and physical needs, is needed (Coulter and Oldham 2016). This underscores the essence of person‐centered care, which is empathic and compassionate, ensuring that individuals feel they are the central focus and that their unique needs are being met (Calisi et al. 2016).

To shift the focus towards more person‐centered care, a crisis intervention called Patient‐Initiated Brief Admission (PIBA) has been developed (Helleman et al. 2014a). It is designed to increase patient autonomy, care participation, and self‐awareness, thereby increasing the patient's ability to identify early signs of deterioration in their well‐being, which should ultimately reduce the need for and shorten the duration of inpatient care (Eckerstrom et al. 2019; Strand and von Hausswolff‐Juhlin 2015). PIBA encourages patients to be viewed as experts in their own experience, inviting them to partner in their own care planning (Eckerstrom et al. 2019). PIBA is therefore founded upon a mutual agreement (called PIBA contract or PIBA agreement) between the patient and their care provider, outlining the conditions and content of future admissions, should the patient wish to seek care (Eckerstrom et al. 2022). Healthcare providers cannot override a patient's request to be admitted; instead, patients are given the opportunity to make their own decisions regarding their need for inpatient care through a phone call to the ward, ensuring that care is easily accessible whenever necessary (Strand and von Hausswolff‐Juhlin 2015). In previous studies, different terms for PIBA have been used, such as self‐admission (Strand, Gustafsson et al. 2017), brief admission (Helleman et al. 2014a), patient‐controlled hospital admission (Thomsen et al. 2018), and open borders programme (Mortimer‐Jones et al. 2016). Reaching a consensus on a definition may serve as a catalyst for the consolidation and refinement of its content and structure, potentially enhancing the generalizability of individual studies. PIBA has been suggested as an appropriate name that accurately reflects the intervention's goals (Eckerstrom et al. 2022). The duration of a PIBA admission varies depending on the setting and patient group. For example, 1–3 days for patients with emotional instability and self‐harm (Westling et al. 2019) and 1–7 days for patients with severe mental disorders (Ellegaard et al. 2020) and anorexia nervosa (Strand et al. 2020). The main focus of PIBA is on nursing activities, and it is led by nurses who are responsible for admissions and discharges (Eckerstrom et al. 2022; Enoksson et al. 2022). PIBA for adolescents follows the same overall approach as it does for adults, and adolescents have the choice to stay with or without their parents, unlike traditional care where parents must always accompany their children (Lindkvist, Westling, Eberhard et al. 2021). Patients with a severe mental disorder are offered a PIBA contract if deemed relevant by clinic professionals, based on criteria such as prolonged illness duration, frequent admissions, extended admission periods, and/or a history of coercive measures (Ellegaard et al. 2020).

In recent years, several studies have investigated the effectiveness of PIBA as an intervention within psychiatric care with varying results. A Norwegian longitudinal study including 74 patients with various severe mental disorders and a total of more than 500 admissions reported that the five‐day limit could be maintained in 92.5% of PIBA admissions, with 90% of patients rating themselves as satisfied and more than 90% recommending PIBA to others (Nyttingnes, Saltyte Benth, and Ruud 2021). In a randomised controlled trail (RCT), also conducted in Norway, PIBA showed no significant short‐term effects on the following questionnaires: the Patient Activation Measure (focusing on patient knowledge, skill, and confidence in self‐management) and the Recovery Assessment Scale (personal confidence and hope, willingness to ask for help, goal and success orientation, reliance on others, and degree of domination by symptoms) (Moljord et al. 2016). However, Westling et al. (2019) conducted an RCT in Sweden involving patients with self‐harm and risk of suicide and demonstrated that although patients with access to PIBA had a similar total number of inpatient days compared to the control group. A register‐based study involving patients with borderline personality disorder found that PIBA was associated with a significant reduction in the length of individual hospital stays, although it did not significantly reduce the overall number of inpatient care days (Eckerstrom et al. 2024). An analysis of healthcare utilisation conducted also in Sweden investigated PIBA for eating disorders in psychiatric care and showed a 67% reduction in the need for regular specialist inpatient treatment (Strand et al. 2021). An observational longitudinal cohort study conducted in child and adolescent psychiatry in Sweden showed a decreased demand for emergency care (Johansson et al. 2023).

Efforts have also been made to elucidate various stakeholders' experiences of PIBA, to better understand its perceived benefits and pitfalls, although to date, studies have focused mostly on patients' and nurses' experiences; few have explored relatives' experiences. Studies have shown that nurses perceive PIBA to be helpful for patients (Arnold, Wardig, and Hultsjo 2022; Eckerstrom et al. 2019; Ellegaard et al. 2017; Lindgren et al. 2023; Lindkvist et al. 2019). Specifically, inpatient nurses have reported that PIBA strengthens staff‐patient relationships and reduces conflicts between the groups (Eckerstrom et al. 2019; Lindgren et al. 2023; Lindkvist et al. 2019). Outpatient nurses report feeling safe in the knowledge that their patients have a safety net to fall back on when the outpatient clinic was closed; however, these nurses argue that the credibility of PIBA is at risk when admission cannot be offered due to lack of space (Arnold, Wardig, and Hultsjo 2022; Lindgren et al. 2023). In fact, when nurses have to deny patients admission due to lack of space, they experience an ethical dilemma and stress because they cannot fulfil their duties to the level to which they aspire (Lindkvist et al. 2019). Nevertheless, nurses have reported that PIBA strengthens patient autonomy and care participation (Eckerstrom et al. 2019; Lindgren et al. 2023), strengthens the nurse's professional role (Lindkvist et al. 2019), and promotes better collaboration between inpatient and outpatient staff (Arnold, Wardig, and Hultsjo 2022; Lindgren et al. 2023).

The few studies that have explored relatives' experiences of PIBA have shown that relatives believe that PIBA helps maintain daily routines and improves family relationships (Hultsjo et al. 2022; Lindkvist et al. 2024). Relatives also express feeling a sense of security when their loved one has access to voluntary admission, reducing their constant worry about their loved one's well‐being and fear that they might self‐harm (Hultsjo et al. 2023; Lantto et al. 2023; Lindkvist et al. 2024). When patients are denied voluntary admission however, relatives feel betrayed by the healthcare system, which damages their trust towards psychiatry (Hultsjo et al. 2023; Lantto et al. 2023; Lindkvist et al. 2024).

As mentioned previously, research on PIBA has predominantly focused on patients' experiences, with studies conducted in various countries including Sweden, Norway, Denmark, the Netherlands, and Australia. Despite the increasing volume of qualitative research exploring this perspective, a comprehensive scientific review of the aggregated patient experiences of PIBA has not yet been conducted. Investigating and synthesising these experiences is crucial to deepen our understanding of PIBA's role for patients in psychiatric care and its efficacy. This understanding can contribute to the improvement of psychiatric services that better meets individual patient needs, ultimately improving care quality.

## 2 Aim

This systematic review aimed to explore the experiences of patient‐initiated brief admission (PIBA) among individuals with mental health disorders.

## 3 Methods

### 3.1 Study Design

A systematic review was conducted to compile the current state of knowledge on patients' experiences of PIBA within psychiatry. This review was guided by the methods described by Polit and Beck (2021) and Bettany‐Saltikov and McSherry (2016), and the updated version of the Preferred Reporting Items for Systematic Reviews and Meta‐Analyses (PRISMA, Data S1) statement (Page et al. 2021).

### 3.2 Data Collection Processes

#### 3.2.1 Eligibility Criteria

Inclusion and exclusion criteria were predetermined to identify articles that met the study's objectives and to exclude irrelevant ones. A useful tool for breaking down one's objectives, identifying keywords, and predetermining inclusion and exclusion criteria is the Population, Exposure, and Outcome (PEO) framework (Bettany‐Saltikov, and McSherry 2016). The PEO framework used in this study is presented in Table 1.

**TABLE 1:** The PEO framework utilised in this systematic review, along with the terms used for database searches.

	Focus areas	Subject terms used in database searches
Population	Patients with access to PIBA	Patient* OR User* OR Adolescent*
Exposure	Patient‐initiated brief admission	Patient initiated brief admission* OR Brief admission* OR Self‐admission* OR Patient‐controlled admission* OR Self‐referral inpatient treatment* OR Self‐referral admission* OR Open Borders programme* OR
Outcome	Experiences	Experience* OR View* OR Perspective* OR Use*

Selection criteria were scientific original articles written in English, that were both peer‐reviewed, and reviewed by an ethical board. Moreover, since the objective was to highlight patient experiences, only qualitative articles were included. The literature review was not restricted by year of publication or participant age. Unavailability of a full text article was also not considered sufficient for exclusion.

#### 3.2.2 Information Sources and Search Strategy

Relevant articles were initially identified through a systematic database search of PubMed, CINAHL (Cumulative Index to Nursing and Allied Health Literature), and PsychInfo using the subject search terms presented in Table 1. The combination of subject search terms with a free‐text search has been shown to yield more precise results (Bettany‐Saltikov and McSherry 2016). In PubMed, no limitations were used, while CINAHL and PsychInfo “Peer‐reviewed” were selected as a limitation. The searches were conducted in 2023. Once eligible articles were identified through the systematic database search, an additional manual article search was conducted by going through the reference lists of the included articles, as recommended by Bettany‐Saltikov and McSherry (2016). The final search strategy and the results of the systematic search are presented in Appendix S1.

#### 3.2.3 Article Selection Process

**FIGURE 1:**
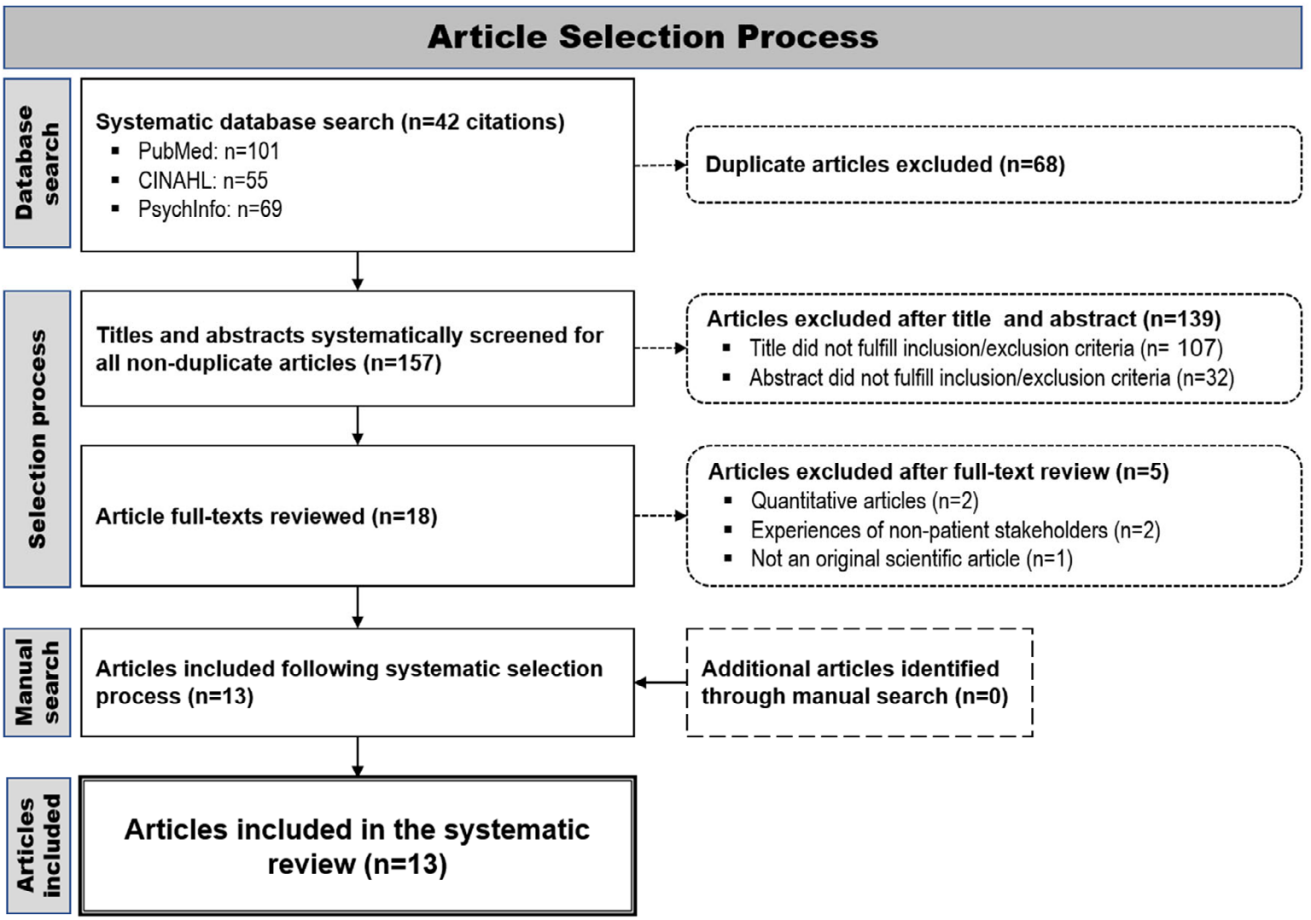
Article selection process.

A total of 225 citations were identified following the systematic database search. After duplicate articles (*n* = 68) identified across multiple databases were excluded, 157 articles remained and were screened according to Bettany‐Saltikov and McSherry (2016). Articles where titles (*n* = 107) or abstracts (*n* = 32) did not meet the predetermined inclusion and exclusion criteria were immediately excluded. The remaining articles (*n* = 18) were read in their entirety. Of these, 5 articles did not meet inclusion criteria and were thus excluded for the following reasons: quantitative methods used (*n* = 2), article described the experiences of non‐patient stakeholders (*n* = 2), or not an original scientific article (*n* = 1). In total, 13 eligible articles identified through the systematic database search met all inclusion criteria. No further articles that met all inclusion criteria emerged in the additional manual article search. Therefore, a total of 13 articles were included in this systematic review (Appendix S2). The article selection process is illustrated in Figure 1.

During the article selection process, the review template “Assessment of Studies with Qualitative Methodology”, designed by the Swedish Agency for Health Technology Assessment and Assessment of Social Services (SBU), was utilised to assess the methodological limitations in the selected articles (SBU 2022). His template is specifically crafted for the systematic review of methods and interventions in healthcare (SBU 2022). According to the template, the selected articles exhibited either low or moderate limitations (as presented in Appendix S2); therefore, no articles were excluded. Additionally, the relevance of the studies was independently reviewed by EV and JN. The interreviewer agreement was assessed, and both reviewers independently reached the same conclusions regarding the relevance of the articles for full‐text review.

### 3.3 Synthesis Methods

Thematic synthesis was conducted to compile and integrate the results of the selected articles (Thomas and Harden 2008). This method follows three steps, outlined briefly below.

#### 3.3.1 Step 1: Free Line‐By‐Line Coding of Findings of Primary Studies

All meaning units were extracted from the results of the included studies and compiled in Microsoft Word. The co‐authors (EV & JN) subsequently individually coded all meaning units inductively, according to its meaning and content. Each meaning unit had at least one code applied, with similar codes sometimes grouped and sorted under higher‐level parent codes. EV and JN convened after the coding process to ensure coding consistency.

#### 3.3.2 Step 2: Organisation of Codes and Development of Descriptive Themes

The co‐authors (E.V. & J.N.) collaboratively reviewed all codes for similarities and differences and to group them into descriptive themes. When necessary, new codes were generated to capture the meaning of clusters of initial codes.

#### 3.3.3 Step 3: Generation of Analytical Themes

The co‐authors (E.V. & J.N.) met regularly to discuss, analyse, and reflect upon the content of the descriptive themes and their alignment with the study's purpose. This process enabled them to develop main analytical themes and sub‐themes. The remaining co‐authors (J.E. & E.A.S.‐G.) were involved in the final stages of the thematic synthesis to help formulate and define the final analytical themes.

## 4 Results

This systematic review reveals insights from 13 qualitative studies originating from Sweden (*n* = 7), the Netherlands (*n* = 2), Norway (*n* = 2), Australia (*n* = 1), and Denmark (*n* = 1) and includes a total of 186 patients. The qualitative data from these articles were systematically and thematically synthesised, yielding three overarching analytical themes and seven corresponding sub‐themes related to patient‐initiated brief admission (PIBA). These are outlined in Table 2 and are further described in the subsequent text.

**TABLE 2:** Analytical themes and sub‐themes identified through thematic synthesis of 13 qualitative studies, including which articles are included in each sub‐theme.

Analytical themes	An internal struggle: PIBA provides comfort and a sense of security, yet challenges persist			Time out: More than just a break		Long‐term benefits of PIBA: Strengthened family and self‐relationships	
Sub‐themes	Experiencing a warm welcome upon admission	Security in knowing that help is readily available	Help‐seeking barriers	Getting respite when everyday life becomes too difficult to handle	Synchronisation of healthcare and everyday life	Strengthening family and significant other relationships	Fostering self‐growth and responsibility
**Articles included in each sub‐theme**							
1. Eckerstrom et al. (2020)	x	x	x	x			
2. Ellegaard et al. (2020)	x	x	x	x			x
3. Enoksson et al. (2022)	x	x		x	x	x	
4. Helleman et al. (2014b)	x	x	x	x	x		x
5. Helleman et al. (2016)			x				
6. Helleman et al. (2018)	x	x	x	x	x		x
7. Lindkvist, Westling, Eberhard et al. (2021)	x	x	x	x	x	x	x
8. Lindkvist, Westling, Liljedahl et al. (2021)	x	x	x	x	x	x	x
9. Mortimer‐Jones et al. (2019)	x	x		x			x
10. (Olsø et al. 2016)		x	x	x	x		
11. Rise et al. (2014)					x		x
12. Strand, Bulik et al. 2017		x	x	x	x	x	x
13. Strand, Gustafsson et al. (2017)			x				

### 4.1 An Internal Struggle: PIBA Provides Comfort and a Sense of Security, Yet Challenges Persist

PIBA stands at the crossroads of contemporary psychiatric care, offering refuge to those in turmoil while presenting its own set of challenges. The following sections delve into the dichotomies of this approach—its provision of solace and the hurdles that patients face. Initial interactions with healthcare staff, the importance of help availability, and barriers to accessing care will be examined, shedding light on the complexities of support and the persistent obstacles in the patient's path to recovery.

#### 4.1.1 Experiencing a Warm Welcome Upon Admission

Patients felt that a warm welcome from healthcare staff greatly impacted their experience of the care they received during PIBA (Eckerstrom et al. 2020; Ellegaard et al. 2020; Enoksson et al. 2022; Helleman et al. 2014b, 2018; Lindkvist, Westling, Eberhard et al. 2021; Lindkvist, Westling, Liljedahl et al. 2021; Mortimer‐Jones et al. 2019). They also reported that the initial contact was crucial, and a warm welcome was described as fundamental in the their care experience (Helleman et al. 2018). Encountering kindness, encouragement, and respect could alleviate severe emotions such as self‐harm urges or suicidal thoughts. Moreover, this welcoming approach helped create order in patients' emotional chaos (Helleman et al. 2014b) and allowed them to feel supported, ensuring they did not have to struggle with their difficulties alone (Ellegaard et al. 2020).

Eckerstrom et al. (2020) reported that patients felt comfort when receiving praise from staff for initiating contact early to avoid further deterioration in their condition. It made them feel validated and taken seriously, which contrasted greatly to their previous experiences of early care‐seeking (outside of PIBA), where they felt that they were not taken seriously by healthcare staff. Patients described a consistently warm and welcoming environment during PIBA, where conversations with staff not only fostered a sense of security but also enhanced their motivation to take responsibility for their own well‐being (Enoksson et al. 2022).

#### 4.1.2 Security in Knowing That Help is Readily Available

PIBA fostered a sense of security among patients (Eckerstrom et al. 2020; Ellegaard et al. 2020; Enoksson et al. 2022; Helleman et al. 2014b, 2016; Lindkvist, Westling, Eberhard et al. 2021; Lindkvist, Westling, Liljedahl et al. 2021; Mortimer‐Jones et al. 2019; Olsø et al. 2016; Strand, Bulik et al. 2017). The assurance of being able to contact the unit at all hours of the day was highlighted as a particularly important contributing factor (Eckerstrom et al. 2020; Ellegaard et al. 2020; Helleman et al. 2016; Lindkvist, Westling, Eberhard et al. 2021; Mortimer‐Jones et al. 2019). They reported that knowing that PIBA was readily accessible to them and that they would receive a warm welcome if care were sought had a calming effect on them (Eckerstrom et al. 2020). Interestingly, this sense of reassurance extended to patients who did not actively use the intervention, with the mere availability of PIBA acting as a safety net and enhancing their sense of security (Lindkvist, Westling, Liljedahl, et al. 2021; Strand, Bulik et al. 2017).

Patients described that PIBA created increased predictability, which in turn also fostered a sense of security (Helleman et al. 2018; Lindkvist, Westling, Eberhard et al. 2021). They were more willing to handle difficult emotions at home before seeking (Enoksson et al. 2022; Olsø et al. 2016), knowing that if they felt care was needed, they could call the ward to discuss their need for PIBA without feeling judged (Lindkvist, Westling, Liljedahl, et al. 2021). Being asked about their individual needs when seeking admission also made patients feel that staff took interest and were engaged in their care (Lindkvist, Westling, Liljedahl et al. 2021).

#### 4.1.3 Help‐Seeking Barriers

Despite the experienced benefits of PIBA, patients reported encountering difficulties contacting the ward in time to request PIBA (Eckerstrom et al. 2020; Ellegaard et al. 2020; Helleman et al. 2014b, 2016; Lindkvist, Westling, Eberhard et al. 2021; Lindkvist, Westling, Liljedahl et al. 2021; Strand, Bulik et al. 2017), particularly when their anxiety arose suddenly and escalated rapidly (Ellegaard et al. 2020; Helleman et al. 2014b). Previous negative experiences with inpatient care and emergency visits also influenced patients' decisions to seek PIBA (Lindkvist, Westling, Eberhard et al. 2021; Lindkvist, Westling, Liljedahl et al. 2021). They found the responsibility of determining when to request PIBA both unfamiliar and difficult (Lindkvist, Westling, Eberhard et al. 2021; Strand, Bulik et al. 2017). Assessing the need for PIBA was described as difficult, with their questioning whether thoughts of self‐harm or fatigue were sufficient reasons to request an admission. This led some to delay help‐seeking, resulting in a deterioration of their condition (Lindkvist, Westling, Liljedahl et al. 2021). Patients also reported sometimes feeling undeserving of a place on the ward or that someone else might be more deserving, which also created hesitancy in seeking PIBA (Helleman et al. 2016, 2018; Lindkvist, Westling, Eberhard et al. 2021; Lindkvist, Westling, Liljedahl et al. 2021; Strand, Bulik et al. 2017).

The care environment itself and fellow patients could also sometimes create resistance to seeking PIBA (Eckerstrom et al. 2020; Helleman et al. 2014b; Lindkvist, Westling, Eberhard et al. 2021; Lindkvist, Westling, Liljedahl et al. 2021; Strand, Bulik et al. 2017; Strand, Gustafsson et al. 2017). Patients occasionally found it challenging to maintain healthy personal boundaries with fellow patients while seeking PIBA, which risked them focusing their attention on others rather than themselves (Helleman et al. 2014b). They also experienced difficulty being cared for in the same ward as acutely ill patients, exposing them to the emotional outbursts and self‐harming behaviours of other patients (Lindkvist, Westling, Eberhard et al. 2021).

The absence of an available bed for PIBA was also described as a significant challenge (Eckerstrom et al. 2020; Lindkvist, Westling, Eberhard et al. 2021; Lindkvist, Westling, Liljedahl et al. 2021; Strand, Bulik et al. 2017; Strand, Gustafsson et al. 2017). Having the courage to take the initiative to request PIBA was difficult enough; therefore, finding out that no beds were available was described as disappointing and could lead to loss of trust in the intervention itself (Eckerstrom et al. 2020). Lindkvist, Westling, Liljedahl et al. (2021) described how patients found that their daily lives were disrupted when they had prepared for admission but could not be admitted due to lack of beds.

Patients also reported that their experience of PIBA was undermined when staff lacked knowledge or held negative attitudes towards the intervention (Eckerstrom et al. 2020; Helleman et al. 2014b; Lindkvist, Westling, Eberhard et al. 2021; Lindkvist, Westling, Liljedahl et al. 2021; Strand, Bulik et al. 2017; Strand, Gustafsson et al. 2017). They found it challenging to interact with staff with limited knowledge of PIBA (Eckerstrom et al. 2020) and having to explain the concept to staff created feelings of uncertainty among patients (Helleman et al. 2014b). Furthermore, inconsistent information from different members of staff made some patients feel more ambivalent about admitting themselves (Strand, Gustafsson et al. 2017). Finally, patients expressed fear that PIBA could inhibit their independence due to a continued reliance on inpatient care to manage their condition (Olsø et al. 2016; Strand, Bulik et al. 2017).

### 4.2 Time out: More Than Just a Break

The following theme explores the dynamics of using PIBA for temporary retreats in mental healthcare. Patients find these interventions serve a dual purpose. First, they provide vital respite for individuals overwhelmed by daily life pressures, offering an opportunity for recuperation and reflection. Second, PIBA enables patients to seamlessly continue with their daily routines while using mental healthcare services, ensuring continuity in their personal and professional lives.

#### 4.2.1 Getting Respite When Everyday Life Becomes Too Difficult to Handle

Patients experienced PIBA as a respite from everyday life, particularly when things became unmanageable (Eckerstrom et al. 2020; Ellegaard et al. 2020; Enoksson et al. 2022; Helleman et al. 2014b; Lindkvist, Westling, Eberhard et al. 2021; Olsø et al. 2016; Strand, Bulik, et al. 2017). This break allowed patients to temporarily rid themselves of the daily demands of life, providing them with an opportunity to focus on what was not working in their lives (Ellegaard et al. 2020). They highlighted that rest and recovery were particularly important components of PIBA (Ellegaard et al. 2020; Helleman et al. 2014b; Olsø et al. 2016). Patients described that by being freed of everyday chores such as cooking and cleaning, provided them with the opportunity to engage in activities that contributed to their improved well‐being (Lindkvist, Westling, Eberhard et al. 2021).

In fact, patients reported that PIBA helped them break the negative spiral of worsening symptoms and negative thoughts (Ellegaard et al. 2020; Enoksson et al. 2022; Helleman et al. 2014b; Lindkvist, Westling, Eberhard et al. 2021; Lindkvist, Westling, Liljedahl et al. 2021; Mortimer‐Jones et al. 2019; Olsø et al. 2016) and provided them with a more dignified alternative than losing control and having life interrupted by a crisis (Lindkvist, Westling, Eberhard et al. 2021). They also reported that PIBA helped them prevent self‐harming behaviour (Enoksson et al. 2022; Lindkvist, Westling, Eberhard et al. 2021) and suicide attempts (Lindkvist, Westling, Eberhard et al. 2021).

Olsø et al. (2016) also reported that patients felt that a healthcare environment characterised by trust and respect resulted in an increased sense of freedom, which was important for their effective recovery. PIBA helped them establish better routines in their diet and circadian rhythm (Helleman et al. 2014b, 2018). The routines and structure of the ward were found to facilitate the patient's ability to resume healthy routines that they could more easily maintain, even after discharge (Ellegaard et al. 2020).

#### 4.2.2 Synchronisation of Healthcare and Everyday Life

Patients experienced PIBA as minimally disruptive, as it allowed them to retain aspects of their daily life, such as work, school, and close relationships, with few interruptions despite being hospitalised (Enoksson et al. 2022; Helleman et al. 2014b, 2018; Lindkvist, Westling, Eberhard et al. 2021; Lindkvist, Westling, Liljedahl et al. 2021; Olsø et al. 2016; Rise et al. 2014). Without compulsory care, patients sought care on their own terms, which also allowed them to influence the total number of inpatient days. This was appreciated by patients, leading them to feel in greater control of their lives. It also gave them better opportunities to maintain employment or continue their studies (Enoksson et al. 2022). Furthermore, patients reported that using PIBA helped them to maintain their social roles such as parenthood, educational activities, and work (Helleman et al. 2018).

Being allowed to keep their personal belongings during their admission was described as positive by patients, giving them an increased sense of integrity and independence (Lindkvist, Westling, Eberhard et al. 2021). They also appreciated the freedom to come and go from the ward as they pleased during their hospitalisation (Lindkvist, Westling, Liljedahl et al. 2021).

### 4.3 Long‐Term Benefits of PIBA: Strengthened Family and Self‐Relationships

The final theme describes how patients express that PIBA, offers not just short‐term benefits during their admission, but also long‐term positive impacts on their relationships, both with family members and themselves.

#### 4.3.1 Strengthening Family and Significant Other Relationships

Patients felt that PIBA impacted their relationships with their relatives (Enoksson et al. 2022; Lindkvist, Westling, Eberhard et al. 2021; Lindkvist, Westling, Liljedahl et al. 2021; Strand, Bulik et al. 2017). According to patients, knowing where to seek help should their condition deteriorate, provided both them and their relatives a sense of relief (Lindkvist, Westling, Eberhard et al. 2021). In fact, they described having previously felt guilty that their relatives were concerned for their safety; therefore, PIBA was believed to help lessen the burden of their condition on their relatives (Lindkvist, Westling, Eberhard et al. 2021; Lindkvist, Westling, Liljedahl et al. 2021; Strand, Bulik et al. 2017). Patients also felt that they became more open to receiving advice from relatives about seeking help when their condition worsened. This, in turn, led them to feel less of a burden on their relatives (Lindkvist, Westling, Liljedahl et al. 2021).

Moreover, controlling their own admission was perceived by patients to make it easier for them to maintain relationships with their families. This opportunity led patients to feel less powerless and distressed (Enoksson et al. 2022). Finally, an individual patient followed over an extended period and described that PIBA helped strengthen her communication skills and social relationships, which she viewed as an important aspect of her recovery (Helleman et al. 2018).

#### 4.3.2 Fostering Self‐Growth and Responsibility

Patients described having grown in their own relationship with themselves after having access to PIBA (Ellegaard et al. 2020; Enoksson et al. 2022; Helleman et al. 2014b, 2018; Lindkvist, Westling, Eberhard et al. 2021; Lindkvist, Westling, Liljedahl et al. 2021; Mortimer‐Jones et al. 2019; Olsø et al. 2016; Rise et al. 2014; Strand, Bulik et al. 2017). They experienced an increased sense of responsibility for their own well‐being and their lives (Helleman et al. 2014b; Lindkvist, Westling, Eberhard et al. 2021; Lindkvist, Westling, Liljedahl et al. 2021; Mortimer‐Jones et al. 2019; Olsø et al. 2016). According to Helleman et al. (2018), despite patients describing having this increased sense of responsibility as something new and somewhat unfamiliar, it was nonetheless considered positive and an opportunity to develop self‐reflection.

Moreover, when healthcare staff exhibited trust in the patient's abilities, patients found that it strengthened their self‐confidence and self‐awareness. Patients felt they became more self‐empathetic as they listened to their own needs, which helped them admit themselves early to avoid self‐harm or symptom deterioration (Ellegaard et al. 2020; Enoksson et al. 2022; Helleman et al. 2014b; Lindkvist, Westling, Eberhard et al. 2021; Lindkvist, Westling, Liljedahl et al. 2021). Patients believed that they understood themselves and their problems better when given an increased sense of freedom (Ellegaard et al. 2020) and were able to shoulder a greater sense of responsibility (Enoksson et al. 2022). They were more aware of early signs of deterioration and were able to act upon these in time (Enoksson et al. 2022). Similarly, Lindkvist, Westling, Eberhard et al. (2021) found that patients believed that PIBA contributed to their maturation and ability to actively seek and receive help in a timely manner. Finally, patients described that the lessons they learned through PIBA not only strengthened their independence, reduced self‐harming behaviour, and decreased the time they spent in inpatient care, but also improved their quality of life (Mortimer‐Jones et al. 2019).

## 5 Discussion

This systematic review revealed that PIBA has significantly influenced patient engagement in psychiatric care, allowing them to autonomously decide when to seek care, which represents a notable shift from past practices. Patients' experiences with PIBA include rapid access to healthcare, fostering a sense of security, contributing to their recovery, and enabling them to maintain day‐to‐day activities and relationships with minimal disruption. Additionally, they report improvement not only in their self‐perception but also in their relationships with others. However, despite these benefits, patients also identify challenges with PIBA. Addressing these challenges is crucial to refine and adapt the method to better meet patient needs. The identified advantages and disadvantages of PIBA are further discussed below across three key areas: its role as a treatment, healthcare accessibility through PIBA, and its impact on recovery and daily life.

### 5.1 PIBA as Nursing Intervention

Patients described PIBA as a helpful intervention that played a significant role in their overall experience and ultimate recovery. They voiced a change in staff attitudes during admission, from previously feeling that they were not taken seriously by staff, to feeling acknowledged and encouraged when they initiated admission. Interactions with healthcare professionals characterised by a warm welcome and daily conversations are important and considered particularly instrumental in alleviating suffering and preventing self‐harm. These results align with previous quantitative findings by Eckerstrom et al. (2022), which demonstrated a significant reduction in anxiety and depression and increased health‐related quality of life. The importance of good and respectful interactions with healthcare staff in psychiatric care is also underscored by Stewart et al. (2015), whose research indicated that a supportive attitude contributed to an overall favourable care experience. This is consistent with another quantitative study where patients' experiences of the quality of care directly correlated with staff interaction and their communication abilities (Keller et al. 2013). Given the suggested benefits of PIBA for patients with self‐harming behaviour, it might be a suitable intervention option for them, one that should be offered more systematically across this patient group.

Potential mechanisms behind the perceived shift in attitudes among healthcare professionals in the present study include the application of the theoretical concepts underpinning PIBA. Eckerström (2022) highlighted that patient participation, shared decision‐making, and patient autonomy are the foundation of PIBA. These concepts align with the framework of person‐centred care, which aims to shift the focus from the patient as only a medical entity, to the person as a whole, emphasising that the patient's life history, needs, and wishes should be acknowledged (Coulter and Oldham 2016). Samuelsen, Moljord, and Eriksen (2016) discussed the underlying approach of PIBA and concluded that the role of healthcare professionals is to provide a supportive and empowering environment for patients. This fosters hope, enabling them to take control of their own lives. It involves helping them become self‐empowered and tailoring their goals to their specific circumstances. This responsibility is particularly crucial for patients with a longstanding history of mental health disorders (Samuelsen, Moljord, and Eriksen 2016).

To address some of the improvement needs identified in the present study, the following issues were noted: (1) patients find it difficult to assess when it is appropriate to seek PIBA; (2) patients feel undeserving of using PIBA‐designated rooms; and (3) patients perceive that not all staff have adequate knowledge about PIBA and some still hold negative attitudes towards it. Nurses working with PIBA have previously described that shifting towards more person‐centred care can be challenging (Eckerstrom et al. 2019). To counter these issues, nursing supervision for healthcare staff is suggested (Eckerström, 2022). Promoting regular nursing supervision in wards that have implemented PIBA could better support healthcare professionals in various clinical situations, such as managing patient crises, communicating effectively with patients, and making informed decisions about patient care. This supervision can help develop knowledge and competence in their own practice and assist staff in changing their behaviours and attitudes towards patients (Eckerström, 2022).

### 5.2 Healthcare Accessibility Through PIBA

The ability to access the ward at all hours of the day is considered a particularly important component of PIBA. Patients describe that knowing PIBA is an available option, not only has a calming effect but also contributes to them enduring symptoms at home longer than they would have in the past. Even those who have not yet utilised PIBA describe that knowing care is available when needed creates a sense of security. This suggests that PIBA, along with rapid access to care, provides reassurance and encourages patients to refrain from seeking immediate care. Instead, they feel secure enough to manage difficult feelings outside of a healthcare setting. Conversely, nurses have previously expressed fear that patients with access to PIBA might over‐utilise care (Lindkvist et al. 2019). However, this fear was soon alleviated when nurses began working with PIBA (Lindkvist et al. 2019). Quantitative studies examining the impact of PIBA on healthcare utilisation have shown that patients, in fact, did not overutilize care and demonstrated a reduction in the use of round‐the‐clock inpatient care (Sigrunarson et al. 2017; Thomsen et al. 2018).

A challenge identified by patients in the studies included in this review is that they are occasionally denied admission due to bed shortages. This leads not only to disappointment but, more seriously, can decrease the patients' trust in the intervention. Nurses have also expressed frustration with bed shortages (Lindgren et al. 2023; Lindkvist et al. 2019), making them feel inadequate, stressed, and worried when forced to deny patients PIBA due to a lack of available beds (Lindkvist et al. 2019).

Care‐seeking patients, especially those with emotional instability, often face resistance from emergency care staff, leading them to feel perceived as a burden and hesitant to seek further healthcare (Vandyk et al. 2019). However, patients enrolled in PIBA experience a different reception from healthcare staff compared to regular psychiatric admission procedures through emergency units. Nurses report that PIBA transforms the admission process from a time‐consuming struggle into a time‐limited break with direct communication between the assigned nurse and the patient, reducing misunderstandings that can sometimes arise when communications are filtered through several nurses and physicians (Eckerstrom et al. 2019).

### 5.3 Recovery and Ability to Maintain Day‐to‐Day Activities and Relationships

A central finding of this systematic review is that PIBA provides a respite from the demands of everyday life, facilitating patient recovery. By allowing patients to step away from daily responsibilities, PIBA enables them to concentrate on their recovery and subsequently resume their everyday lives. PIBA promotes a balance between activities and rest (Eckerström, 2022), and recovery is sometimes described as simply having the freedom to exist without additional expectations or pressures (De Ruysscher et al. 2020). A significant aspect of PIBA is that patients perceive inpatient care as less intrusive on their everyday lives, which is crucial for their recovery. Westling et al. (2019) conducted an RCT in Sweden which showed that patients with access to PIBA had a similar total number of inpatient days as those receiving standard care. This finding suggests that while PIBA provides predictability and a sense of control, it does not necessarily reduce the overall need for inpatient care. However, PIBA offers respite by giving individuals the opportunity to step back from their everyday challenges, while simultaneously enabling them to maintain important aspects of their daily lives. While PIBA does not reduce the total number of inpatient days compared to standard care, the set number of inpatient days offers a sense of predictability. This predictability makes it easier for individuals to plan their lives, as they can anticipate and prepare for their time in care. This structure aligns with the ultimate goal of PIBA: to provide individuals with a sense of control over not only their care but also their lives (Liljedahl et al. 2017).

Finally, this review also found that PIBA promotes better relationships between patients and their relatives. Knowing that help is available through PIBA allows patients to feel less of a burden on their relatives. Similar experiences have been expressed by relatives; they report that PIBA improves the patients' ability to maintain everyday routines and strengthen their family relationships (Hultsjo et al. 2023). Patients also report that PIBA has strengthened their ability to reclaim independence and increased their quality of life.

### 5.4 Methodological Strengths and Limitations

This systematic review exhibits methodological strengths and limitations that are common in systematic reviews. Insights from 202 patients experiencing PIBA across five different countries, provided a rich dataset, A notable strength of this review is its comprehensive and methodical approach, supported by adherence to well‐established frameworks such as PRISMA and the PEO framework, ensuring a structured approach to literature search and analysis (Siddaway, Wood, and Hedges 2019). The employment of a review template from the Swedish Agency for Health Technology Assessment and Assessment of Social Services for quality assessment further adds rigour and credibility to the review findings (Munn et al. 2020).

However, there are inherent limitations in this approach. A significant issue is the risk of bias in the included studies, a common challenge in systematic reviews. This bias, arising from limitations in study design, conduct, or analysis, can impact the overall conclusions of this review (Munn et al. 2020). Additionally, the focus on qualitative articles introduces specific challenges, including complex data analysis and the influence of researchers' subjectivity, potentially affecting the synthesis process and interpretation of findings. Moreover, the included studies originate primarily from Northern Europe (Scandinavia and the Netherlands) and Australia. The absence of studies from other continents, particularly those from diverse socioeconomic backgrounds, limits the generalizability of our findings. Therefore, more studies from diverse regions and socioeconomic contexts are needed to enhance the robustness and generalizability of our conclusions.

## 6 Conclusions

PIBA represents a novel and promising psychiatric intervention, offering patient elements they have historically lacked: a warm welcome, dignity, and a sense of security. PIBA not only positively impacts patients but also extends benefits to their relatives by fostering a functional everyday life. The demeanour of staff on the ward and their attitude towards PIBA play a crucial role in shaping the patient experience. A warm welcome, being seen, and being listened to are essential for a positive patient experience, underscoring the importance of staff being well‐informed about the intervention and supporting it. Significantly, PIBA holds the potential to alter patients' perceptions of psychiatric care and access to it, and most importantly, it can change their view of themselves as autonomous individuals who are ultimately responsible for their own well‐being. It is important to note that many of the beneficial elements highlighted within the themes and sub‐themes, such as improved access to care, a welcoming environment, dignity, a sense of security, and positive staff attitudes, are equally relevant to patients not enrolled in PIBA.

## 7 Relevance For Clinical Practice

This systematic review offers essential insights into healthcare professionals working clinically with PIBA. As the results highlight, staff knowledge of the intervention significantly influences patients' experiences with PIBA, and this review contributes to broadening that knowledge base. Additionally, it provides an overview of the current state of published literature regarding patients' experiences with PIBA in various contexts. The review also identifies several challenges and persistent barriers to the implementation of PIBA, including variability in staff knowledge, instances where PIBA rooms are fully occupied, and some patients' uncertainty about when to utilise PIBA. However, these barriers can be effectively addressed with adequate knowledge and understanding. Therefore, this systematic review delivers person‐centred insights crucial for healthcare systems that are considering implementing PIBA in their settings.

## Author Contributions

J.E., E.V., and J.N. jointly designed the study. E.V., and J.N. were responsible for the data collection and analyses. J.E. reviewed the data collection and analysis and prepared the initial draft in English in collaboration with E.A.S.‐G. All authors contributed to the final version of the manuscript.

## Conflicts of Interest

The authors declare no conflicts of interest.

## Registration and Protocol

In conducting this systematic review, we aimed to comprehensively collate and synthesise the literature on PIBA in psychiatric care. This review was not registered on any registry platform, such as PROSPERO or similar databases. While registration is not mandatory, it enhances scientific quality and helps to prevent duplications. In this instance, we acknowledge that not registering with PROSPERO was a limitation. Nevertheless, we adhere to rigorous methodological standards throughout the review process to ensure the integrity and dependability of our findings.

## Supporting information


Appendix S1‐S2

Data S1

## Data Availability

The data that support the findings of this review are derived from publicly available sources. Articles were identified through comprehensive searches of PubMed, CINAHL (Cumulative Index to Nursing and Allied Health Literature), and PsychInfo databases. All references to the included articles are clearly indicated within the text. We have made every effort to ensure transparency and reproducibility within the constraints of data privacy and confidentiality agreements.
