# Supplementary material for: Patient Experiences of Patient‐Initiated Brief Admission in Psychiatric Care: A Systematic Review
Source: Int J Ment Health Nurs. 2024 Oct 27;34(1):e13457. doi: 10.1111/inm.13457 (PMC11771678; doi:10.1111/inm.13457)
Supplement: Supplementary file 1 — Appendix S1‐S2 [file INM-34-0-s002.docx]

# APPENDIX S1. Search Matrix Utilized and Articles Selected for the Systematic Review

| **Database, Search date** | **Search Blocks/Search Terms Used** | **Hits** *(n)* | **Titles Read** *(n)* | **Abstracts Read** *(n)* | **Articles Included** *(n)* | **Articles Selected**  *(see Appendix 2 for more details)* |
| --- | --- | --- | --- | --- | --- | --- |
| PubMed, 2023-12-28 | ("patient*"[All Fields] OR "user*"[All Fields] OR "adolescent*"[All Fields]) AND ("brief admission*"[All Fields] OR "self admission*"[All Fields] OR "controlled admission*"[All Fields] OR ("self referral"[All Fields] AND "admission*"[All Fields]) OR ("self referral"[All Fields] AND ("inpatient s"[All Fields] OR "inpatients"[MeSH Terms] OR "inpatients"[All Fields] OR "inpatient"[All Fields]) AND "treatment*"[All Fields]) OR ("open"[All Fields] AND ("border"[All Fields] OR "bordered"[All Fields] OR "bordering"[All Fields] OR "borders"[All Fields]) AND "programme*"[All Fields]) OR "patient initiated brief admission*"[All Fields]) AND ("experience*"[All Fields] OR "view*"[All Fields] OR "perspective*"[All Fields]OR "use*"[All Fields]) | 101 | 101 | 37 | 13 | 1. Eckerström J, et al., 2020 2. Ellegaard T, et al., 2020 3. Enoksson M, et al., 2022 4. Helleman M, et al., 2014 5. Helleman M, et al., 2016 6. Helleman M, et al., 2018 7. Lindkvist, R-M, et al., 2021a 8. Lindkvist, R-M, et al., 2021b 9. Mortimer-Jones S, et al., 2019 10. Olsø TM, et al., 2016 11. Rise MB, et a., 2014 12. Strand M, et al., 2017a 13. Strand M, et al., 2017b |
| CINAHL, 2023-12-30 | ("patient*" OR "user*" OR "adolescent*") AND ("brief admission*" OR "self admission*" OR "controlled admission*" OR ("self referral" AND "admission*") OR ("self referral" AND ("inpatient s" OR "inpatients" OR "inpatients" OR "inpatient") AND "treatment*") OR ("open" AND ("border" OR "bordered" OR "bordering" OR "borders") AND "programme*") OR "patient initiated brief admission*" ) AND ( "experience*" OR "view*" OR "perspective*" OR "use*") | 55 | 20  (35 duplicates) | 5 | 0 |  |
| PsychInfo, 2023-12-30 | TX ( "patient*" OR "user*" OR "adolescent*" ) AND TX ( "brief admission*" OR "self admission*" OR "controlled admission*" OR ("self referral" AND "admission*") OR ("self referral" AND ("inpatient s" OR "inpatients" OR "inpatients" OR "inpatient") AND "treatment*") OR ("open" AND ("border" OR "bordered" OR "bordering" OR "borders") AND "programme*") OR "patient initiated brief admission*" ) AND TX ( "experience*" OR "view*" OR "perspective*" OR "use*" ) | 69 | 36  (33 duplicates) | 8 | 0 |  |

# APPENDIX S2. Summary Matrix of Qualitative Articles Included in This Systematic Review (n=13)

| **No.** | **Authors** | **Article title** | **Year, Country** | **Journal** | **Aim/s** | **Qualitative Methods Used** | **Summary of Results** | **Methodological Limitations** |
| --- | --- | --- | --- | --- | --- | --- | --- | --- |
| 1. | Eckerström J, Flyckt L, Carlborg A, Jayaram-Lindström N, Perseius K-I | Brief admission for patients with emotional instability and self-harm: a qualitative analysis of patients’ experiences during crisis | 2020, Sweden | Int J Ment Health Nurs | To explore brief admission (BA) in a Swedish inpatient psychiatric facility. | **Data collection:** 15 semi-structured patient interviews  **Data analysis:** thematic analysis | Brief admission (BA) served as a “time-out” when life’s circumstances were tough. Knowing that help was available when needed was considered comforting, allowing patients to feel safe. BA encouraged patients to take responsibility and view their problems from a different perspective. BA was used as a coping strategy when managing emotions, self-harming behavior, and suicide ideations. Staff’s treatment towards patients impacted their experience of BA both positively and negatively. | Low risk for methodological limitations impacted the findings. All 5 domains in SBU Quality assessment tool are well presented and considered. |
| 2. | Ellegaard T, Bliksted V, Mehlsen M, Lomborg K | Feeling safe with patient‐controlled admissions: a grounded theory study of the mental health patients’ experiences | 2020, Denmark | J Clin Nurs | To investigate patients’ experiences in the Danish patient-controlled admission (PCA) program. Specifically, their concerns, attitudes and strategies used. | **Data collection:** field observations (over 2 days) and 26 semi-structured patient interviews  **Data analysis:** grounded theory | By providing faster access to care, PCA helped patients feel safe, thus preventing further symptom deterioration. The support and guidance received during admission by healthcare professionals, patient’s own self-determination, and ability to achieve calmness, all helped contributed the patient’s increased sense of security. Conversely, PCA sometimes led patients to feel uncertain and overlooked by healthcare professionals, which could have undermined their sense of security. | Low risk for methodological limitations impacted the findings. All 5 domains in SBU Quality assessment tool are well presented and considered. |
| 3. | Enoksson M, Hultsjö S, Wärdig RE, Strömberg S | Experiences of how brief admission influences daily life functioning among individuals with borderline personality disorder (BPD) and self-harming behavior | 2022, Sweden | J Clin Nurs | To explore experiences of how BA influences daily life functioning among individuals with BPD and self-harming behavior. | **Data collection:** 16 semi-structured patient interviews  **Data analysis:** content analysis | BA was seen as an intervention that promoted individuals’ self-determination and self-care, and eased their ability to maintain daily routines, employment, and relationships. BA already created a sense of security and was considered a good strategy to help avoid destructive behavior. | Low risk for methodological limitations impacted the findings. All 5 domains in SBU Quality assessment tool are well presented and considered. |
| 4. | Helleman M, Goossens PJJ, Kaasenbrood A, van Achterberg T | Experiences of patients with borderline personality disorder with the brief admission intervention: a phenomenological study | 2014, The Nether-lands | Int J Ment Health Nurs | To describe the lived experiences of patients with BPD with use of the BA intervention. | **Data collection:** 17 in-depth patient interviews  **Data analysis:** phenomenological analysis | The BA’s organization (i.e., care plan, goal setting, admission procedures) as well as the quality of the contact with a nurse, were considered important factors in one’s overall experience of BA, with the latter being most important. BA helped patients overcome crises. And a ”time-out” from daily life, a form of distraction. This gave the patient the opportunity to recover, and embed more structure in their life. Other positive aspects raised were that BA gave patients the opportunity to relax, prevent total loss of control, and improve their autonomy. Some negative experiences described by patients were not feeing seen and feeling lonely. | Low risk for methodological limitations impacted the findings. All 5 domains in SBU Quality assessment tool are well presented and considered. |
| 5. | Helleman M, Goossens PJJ, Kaasenbrood A, van Achterberg T | Brief admissions during prolonged treatment in a case involving borderline personality disorder and posttraumatic stress disorder: use and functions. | 2016, The Nether-lands | J Am Psychiatr Nurses Assoc | To describe how BA may be used during a long-term treatment process. | **Data collection:** a single-case descriptive study with 5 semi-structured interviews (with patient, her husband, psychiatrist, and community psychiatric nurse, and a clinical nurse involved in her care) and chart reviews  **Data analysis:** Key concepts identified, coded, and analyzed to reveal core functions and patterns of BA. | The patient on whom the case study was based believed that BA impacted her in several different ways. She felt that BA helped improve her self-esteem and communication skills. It also helped her better express her thoughts and feelings. This led to her be able to better take her of herself. | Low to moderate risk for methodological limitations impacted the findings. The method for data-analyses is not referred but the steps are presented. The rest of the domains in SBU Quality assessment tool are well presented and considered. |
| 6. | Helleman M, Lundh LG, Liljedahl SI, Daukantaité D, Westling S | Individuals’ experiences with brief admission during the implementation of the brief admission skåne RCT, a qualitative study | 2018, Sweden | Nord J Psychiatry | To investigate participants' experiences with BA during the pilot phase of the BA Skåne Randomized Controlled Trial (BASRCT), to detect possible strengths and limitations of the intervention and gain knowledge to facilitate implementation of BA at other treatment centers. | **Study design:** qualitative  **Data collection:** 8 semi-structured patient interviews  **Data analysis:** thematic analysis | Patients experienced that they received help with basic routines, avoided demands at home, felt safe, and experienced increased predictability. The attitudes of the staff were important for the experience. Experiencing positive and welcoming responses, helpfulness, encouragement, trust, "giving peace", availability, respectfulness, and affirmation through equal treatment was perceived positively. Patients could have negative experiences when staff lacked knowledge about BA, had a negative attitude towards BA, or showed a lack of time and responsibility. Patients felt that BA helped them feel more responsible, feel that others believed in them, and increased self-control. BA could also lead to ambivalence about responsibility and patients feeling they received too much attention. | Low risk for methodological limitations impacted the findings. All 5 domains in SBU Quality assessment tool are well presented and considered. |
| 7. | Lindkvist R-M, Westling S, Eberhard S, Johansson BA, Rask O, Landgren K | ‘A safe place where I am welcome to unwind when I choose to’−experiences of brief admission by self-referral for adolescents who self-harm at risk for suicide: a qualitative study | 2021a, Sweden | Int J Environ Res Public Health | To illuminate adolescents’ experiences of BA, and their suggestions on how BA may be further modified and improved to fit the target group. | **Study design:** qualitative  **Data collection:** 19 semi-structured patient interviews  **Data analysis:** content analysis | BA helped patients feel safe and was helpful to handle impulses to self-harm and to a lesser extent felt like a burden to (loved) relatives. Being welcomed professionally and having access to care with less drama was important to the experience. BA helped patients grow through self-reflection, gave them time for rest and recovery, and a sense of freedom and independence. Negative experiences of BA were receiving insufficient attention, encountering unprofessional behavior, and feeling less prioritized. | Low risk for methodological limitations impacted the findings. All 5 domains in SBU Quality assessment tool are well presented and considered. |
| 8. | Lindkvist R-M, Westling S, Liljedahl SI, Landgren K | A brief breathing space: experiences of brief admission by self-referral for self-harming and suicidal individuals with a history of extensive psychiatric inpatient care | 2021b, Sweden | Issues Ment Health Nurs | To gain knowledge of the meaning of BA for self-harming individuals at high risk of suicide, with histories of extensive psychiatric inpatient care. | **Study design:** qualitative  **Data collection:** 7 semi-structured patient interviews  **Data analysis:** phenomenological-hermeneutic method | BA contributed to patients feeling worthy, regaining control and being welcomed as a unique person. BA gave patients the opportunity to rest and recharge and to have a break from negative events in everyday life. BA was also perceived to provide opportunities for communication with others, developing self-care and taking care of oneself | Low risk for methodological limitations impacted the findings. All 5 domains in SBU Quality assessment tool are well presented and considered. |
| 9. | Mortimer-Jones S, Morrison P, Munib A, Paolucci F,  Neale S, Hellewell A, Sinwan J, Hungerford C | Staff and client perspectives of the Open Borders programme for people with borderline personality disorder | 2019, Australia | Int J Ment Health Nurs | To obtain staff and clients’ perceptions of the clients’ recovery journey subsequent to their admission to the Open Borders programme; determine the clients’ overall perception of the programme; and determine how the staff view their working environment. | **Data collection:** 8 semi-structured patient interviews and 9 semi-structured staff interviews  **Data analysis:** phenomenological analysis | The patients experienced the care environment at Open Borders programme (OBP) as more homely, comfortable and welcoming. Greater focus in care was placed on finding strategies than the usual medical focus. Patients felt it was important to be included in the care. This made them less likely to self-harm and their quality of life increased. Patients experienced support from the healthcare staff. They also appreciated the freedom they received during OBP in being able to maintain their everyday activities. They did not feel rejected to the same extent as they used to in care in general. | Low to moderate risk for methodological limitations impacted the findings. Personal relationship between one author and staff in one of the departments where the study was conducted. The rest of the domains in SBU Quality assessment tool are well presented and considered. |
| 10. | Olsø TM, Gudde CM, Opheim Moljord IE, Evensen GH, Øivind Antonsen D, Eriksen L | More than just a bed: mental health service users’ experiences of self-referral admission | 2016, Norway | Int J Ment Health Syst | To explore the experiences of service users who have had the opportunity to refer themselves for short inpatient stays. | **Data collection:** 42 semi-structured interviews with 28 service users  **Data analysis:** thematic analysis | Self-referral admission (SRA) was experienced by patients as an opportunity to get help quickly and easily when needed. Getting help adapted to personal needs, being able to make the decision to commit oneself without being questioned and having access to a safe place were felt to be important. They also felt that SRA met personal needs. Being able to put themselves in made the pat feel like they were in a decision-making position, this required mutual trust between patients and healthcare staff. SRA also contributed to strengthened self-confidence. | Low to moderate risk for methodological limitations impacted the findings. The gender distribution of the study participants do not match the gender distribution in population of BA. The rest of the domains in SBU Quality assessment tool are well presented and considered. |
| 11. | Rise MB, Evensen GH, Moljord IEO, Rø M, Bjørgen D, Eriksen L | How do patients with severe mental diagnosis cope in everyday life: a qualitative study comparing patients’ experiences of self-referral inpatient treatment with treatment as usual? | 2014, Norway | BMC Health Serv Res | To explore how patients with severe mental diagnosis coped four months after signing a contract for self-referral, as compared to patients receiving treatment as usual. | **Data collection:** 25 semi-structured patient interviews  **Data analysis:** while analysis process was well described (coding with theme development), the analysis method not presented. | Patients who received self-referral inpatient treatment (SRIT) experienced stronger self-confidence in cooping strategies. They also felt that SRIT strengthened cognitive strategies to influence thought patterns  Powerlessness was expressed in both groups but not as profoundly in the group receiving SRIT. Patients who received SRIT experienced a strong sense of being "normal" and living a "normal life" | Moderate risk for methodological limitations impacted the findings. The method for data-analyses is not referred but the steps are still well presented. Also, imbalance in gender between intervention-group and control-group. The rest of the domains in SBU Quality assessment tool are well presented and considered. |
| 12.* | Strand M, Bulik CM, von Hausswolff-Juhlin Y, Gustafsson SA | Self-admission to inpatient treatment for patients with anorexia nervosa: the patient’s perspective | 2017a, Sweden | Int J Eat Disord | To explore patients’ experiences of participating in a PIBAprogram at a specialist eating disorders clinic. | **Data collection:** 16 semi-structured patient interviews  **Data analysis:** content analysis | Self-admission (SA) helped patients experience safety, boost healthy routines, prevent deterioration, obtain motivational support and have interruptions in everyday life. Negative experiences that could come with SA were ambivalence, feeling that other patients could be in greater need of care, being in a triggering environment, feeling that admission presupposes deterioration and fear of having to stay longer than planned in the ward. | Low risk for methodological limitations impacted the findings. All 5 domains in SBU Quality assessment tool are well presented and considered. |
| 13.* | Strand M, Gustafsson SA, Bulik CM, von Hausswolff-Juhlin Y | Self-admission to inpatient treatment in psychiatry: lessons on implementation | 2017, Sweden | BMC Psychiatry | To examine patient experiences of practical considerations during the start-up phase of a PIBAprogram in an eating disorder service. | **Data collection** 16 semi-structured patient interviews  **Data analysis:** content analysis | Patients experienced some "Start up problems" such as problems with getting a bed place and lack of staff continuity. Patients also experienced too little focus on long-term goals. They also felt that self-admission demanded both freedom and responsibility. | Low risk for methodological limitations impacted the findings. All 5 domains in SBU Quality assessment tool are well presented and considered. |

* Interviewed the same informants.
